# Mutagenesis and Functional Analysis of the Pore-Forming Toxin HALT-1 from *Hydra magnipapillata*

**DOI:** 10.3390/toxins7020407

**Published:** 2015-02-03

**Authors:** Yvonne Jing Mei Liew, Wai Tuck Soh, William Febry Jiemy, Jung Shan Hwang

**Affiliations:** Faculty of Applied Sciences, UCSI University, No. 1, Jalan Menara Gading, UCSI Heights, Cheras, 56000 Kuala Lumpur, Malaysia; E-Mails: yvonneljm@gmail.com (Y.J.M.L.); waitucksoh@outlook.com (W.T.S.); willjie90@gmail.com (W.F.J.)

**Keywords:** actinoporins, cytolysin, equinatoxin II, pore-forming protein, mutations, cnidarian

## Abstract

Actinoporins are small 18.5 kDa pore-forming toxins. A family of six actinoporin genes has been identified in the genome of *Hydra magnipapillata*, and HALT-1 (*Hydra* actinoporin-like toxin-1) has been shown to have haemolytic activity. In this study, we have used site-directed mutagenesis to investigate the role of amino acids in the pore-forming *N*-terminal region and the conserved aromatic cluster required for cell membrane binding. A total of 10 mutants of HALT-1 were constructed and tested for their haemolytic and cytolytic activity on human erythrocytes and HeLa cells, respectively. Insertion of 1–4 negatively charged residues in the *N*-terminal region of HALT-1 strongly reduced haemolytic and cytolytic activity, suggesting that the length or charge of the *N*-terminal region is critical for pore-forming activity. Moreover, substitution of amino acids in the conserved aromatic cluster reduced haemolytic and cytolytic activity by more than 80%, suggesting that these aromatic amino acids are important for attachment to the lipid membrane as shown for other actinoporins. The results suggest that HALT-1 and other actinoporins share similar mechanisms of pore formation and that it is critical for HALT-1 to maintain an amphipathic helix at the *N*-terminus and an aromatic amino acid-rich segment at the site of membrane binding.

## 1. Introduction

Actinoporins are a group of potent α-pore forming toxins (α-PFTs) that were first identified in sea anemones [1]. They are low molecular weight proteins with 18.5 kDa and are able to destroy cells containing sphingomyelin, a major component of plasma membrane lipids [2]. Equinatoxin II (EqtII) from *Actinia equina* and sticholysin II (StII) from *Stichodactyla helianthus* are the two best-studied α-PFTs. These α-PFTs were later referred to as actinoporins. The crystal structures of EqtII and StII revealed that actinoporins are single-domain proteins with a compact β-sandwich composed of 12 β-strands aligned in two β-sheets flanked on each side by two short α-helices [3,4,5]. A cluster of exposed aromatic amino acids including a phosphocholine (POC) binding site has been shown to be functionally important for membrane binding. The aromatic amino acids provide initial contact between the protein and the cell membrane while the POC binding site recognizes the headgroup of sphingomyelin in the plasma membrane [6].

The *N*-terminal region comprising 30 residues appears to be the largest amphipathic part of the protein, and this region is the only part of the protein that can undergo conformational changes, whereby it detaches from the core of the protein, without disrupting the fold of the β-sandwich [3]. The flexibility and amphipathic character of this *N*-terminal region are crucial for the mechanism of pore formation as this region is proposed to extend and penetrate into the plasma membrane to form the pore [7]. Pore formation is a multi-step process. The first step is the recognition of sphingomyelin in the lipid membrane. This was supported by the discovery of a POC binding site on the surface of StII that specifically binds to the phosphocholine headgroup of sphingomyelin [5]. After binding to sphingomyelin, the next step of pore formation is the translocation of the *N*-terminal region into the lipid-water interface [8]. The flexibility and the amphipathic nature of this *N*-terminal region allow it to be loosened from the protein and inserted into the lipid membrane [9]. Subsequently, three or four actinoporins oligomerize in the plasma membrane and form an ion conductive pore, leading to the influx of water into the cell [7,10].

Actinoporins were recently found in *Hydra*, a freshwater hydrozoan living in unpolluted lakes and streams [11]. A subsequent study identified six HALT (*Hydra* actinoporin-like toxin) genes in the genome of *Hydra* [12]. These authors also showed that HALT-1 had haemolytic activity similar to, although weaker than, that of equinatoxin II (EqtII) from *Actinia equina.* To further analyze the haemolytic activity of HALT proteins, we have used site-directed mutagenesis to mutate critical residues in the conserved aromatic cluster required for membrane binding and in the amphipathic *N*-terminal α-helix and tested their effect on pore formation. Mutation of residues in these domains strongly reduced haemolytic and cytolytic activity, suggesting that HALT proteins form pores through a similar mechanism to other actinoporins.

## 2. Results

### 2.1. Expression of HALT-1 and Its Mutants

**Figure 1:**
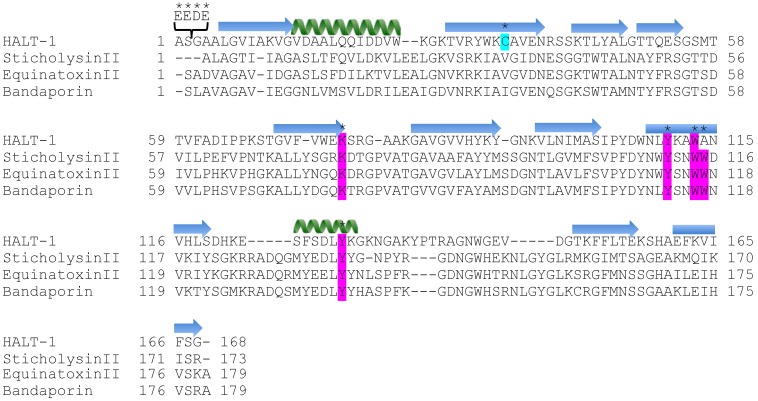
Ten mutations of HALT-1. The amino acid sequence of HALT-1 was aligned with three different actinoporins: sticholysin II (*Stichodactyla* *helianthus*), equinatoxin II (*Actinia equina*) and Bandaporin (*Anthopleura asiatica*). Signal peptides and propeptides for all sequences are excluded in this alignment. Residues with the asterisk marked on top are mutations from insertion, substitution or deletion. Magenta highlights the residues involving in contact with the polar head group of the cell membrane, and light blue highlights the only cysteine present in HALT-1. The 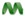 α-helix and 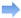 β-strand of HALT-1 are predicted by PSIPRED and marked on the top of the sequence.

**Figure 2:**
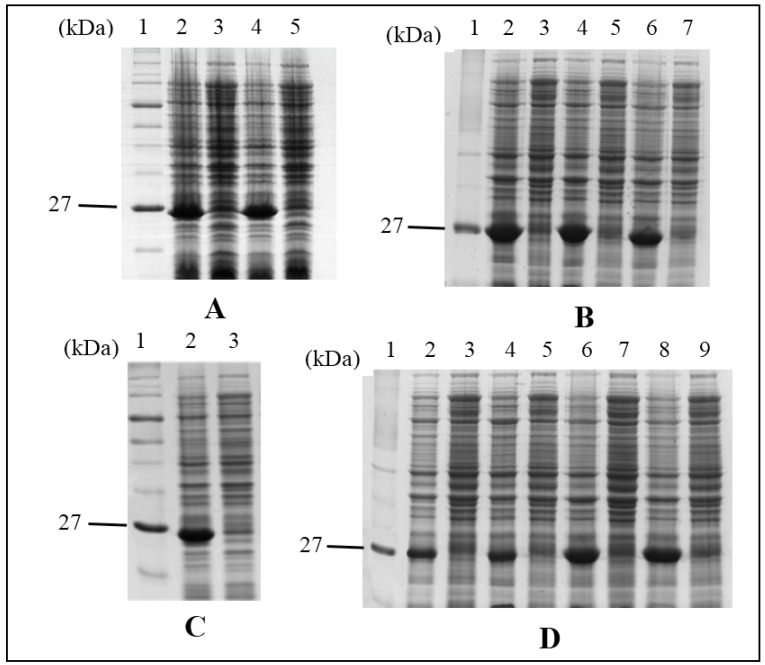
SDS-PAGE image of HALT-1 mutant proteins expressed in BL21(DE3). Expression of mutants was regulated in the presence (induced) or absence (uninduced) of 1 mM IPTG. Lane 1 in all panels is the protein molecular marker. (**A**) lane 2, induced 2E3; lane 3, uninduced 2E3; lane 4, induced 2EE3; lane 5, uninduced 2EE3; (**B**) lane 2, induced 2EED3; lane 3, uninduced 2EED3; lane 4, induced Y110A; lane 5, uninduced Y110A; lane 6, induced K76A; lane 7, uninduced K76A; (**C**) lane 2, induced 2EEDE3; lane 3, uninduced 2EEDE3; (**D**) lane 2, induced A114W; lane 3, uninduced A114W; lane 4, induced 35ΔC; lane 5, uninduced 35∆C; lane 6, induced W113A; lane 7, uninduced W113A; lane 8, induced Y129A; uninduced Y129A.

Ten mutants of HALT-1 were generated by site-directed mutagenesis. Mutants 2E3, 2EE3, 2EED3 and 2EEDE3 altered the length of the *N*-terminal α-helix region by incorporating amino acid(s) successively near the *N*-terminus. Mutants K76A, Y110A, W113A, A114W and Y129A altered amino acids in or near to the conserved aromatic cluster, which is involved in the interaction with cell membrane (Figure 1 and Table 1). To assess whether these mutants have changes in their structures, mutant protein stability was analyzed with SDM (Site-Directed Mutator) and I-Mutant 3.0. Out of these five mutants, SMD’s prediction resulted in four neutral mutations and one stabilizing mutation (Table S1). On the other hand, I-Mutant predicted that three mutants would have minor disturbances (∆∆Gs are greater than −2.0 but less than −0.5) in the local structure while the other two would remain neutral (Table S2). One mutant, 35∆C, was generated by deleting the only cysteine in HALT-1 (Figure 1 and Table 1). All mutants were cloned into the expression vector pET28a and transformed into BL21(DE3). Figure 2 shows the SDS-PAGE images of mutant proteins with the expected protein band at approximately 25 kDa (18.6 kD of HALT-1 mutant and 6.4 kD of vector peptide including 6 histidine tags). Eight out of ten mutants were successfully expressed and purified in this study. Two mutants, A114W and 35∆C, exhibited a low expression level and poor solubility. As a result, sufficient quantities could not be purified for further testing.

**Table 1:** Mutations introduced in HALT-1 amino acid sequence.

Name	Type of mutation	Position	Mutation
2E3	Insertion	3	E
2EE3	Insertion	4	EE
2EED3	Insertion	5	EED
2EEDE3	Insertion	6	EEDE
35∆C	Deletion	35	Removal of cysteine
K76A	Substitution	76	K → A
Y110A	Substitution	110	Y → A
W113A	Substitution	113	W → A
A114W	Substitution	114	A → W
Y129A	Substitution	129	Y → A

### 2.2. Haemolytic Activity of HALT-1 and Its Mutants

**Figure 3:**
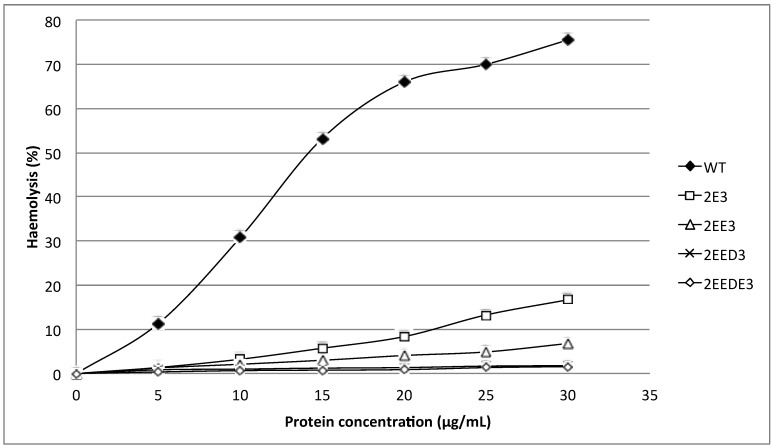
Comparison of wild type (WT) and mutants (2E3, 2EE3, 2EED3 and 2EEDE3) haemolytic activities. A total of 4 amino acids (Glutamic acid-E, Glutamic acid-E, Aspartic acid-D and Glutamic acid-E) were inserted in succession at the *N*-terminus. Mutants generated were of significantly lower haemolytic activity as compared to the wild type.

**Figure 4:**
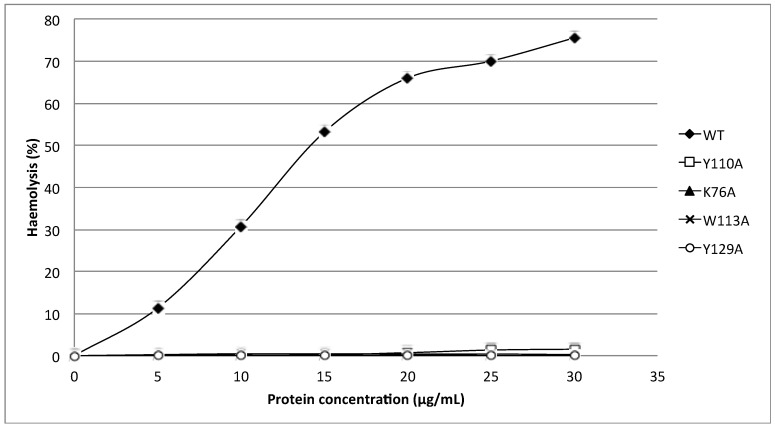
Comparison of wild type (WT) and mutants (Y110A, K76A, W113A and Y129A) haemolytic activities. Three aromatic amino acids and one lysine were substituted individually into alanine (A) and all mutants exhibited a significantly lower haemolytic activity as compared to wild type.

Recombinant HALT-1 was prepared in a serial concentrations of 5, 10, 15, 20, 25, and 30 μg/mL and tested for haemolytic activity using human red blood cells. The results in Figure 3 and Figure 4 show that more than 50% of erythrocytes were completely lysed after 30 min of incubation with 15 μg/mL of recombinant HALT-1, while 80% haemolysis occurred when 30 μg/mL were used. These results are similar to those of Glasser* et al.* [12] and confirm that HALT-1 has haemolytic activity, but is 5–10 times less active than equinatoxin II.

The mutants 2E3, 2EE3, 2EED3 and 2EEDE3, which lengthen the *N*-terminal α-helix, exhibited more than 80% reduction in haemolytic activity compared to wild type HALT-1. At 30 μg/mL, both mutants 2E3 (one-amino acid insertion at the *N*-terminal region) and 2EE3 (two-amino acid insertion) only exhibited ~18% and ~8% of haemolysis, respectively (Figure 3). Further amino acid insertion in mutants 2EED3 and 2EEDE3 (having 3 and 4 amino acid insertions) completely abolished haemolytic activity (below 5%). Mutants with single alanine (A) substitutions in and near the conserved aromatic cluster (K76A, Y110A, W113A and Y129A) caused an almost complete loss of haemolytic activity even at a concentration as low as 5 μg/mL (Figure 4).

### 2.3. Cytolytic Activity of HALT-1 and Its Mutants

**Figure 5:**
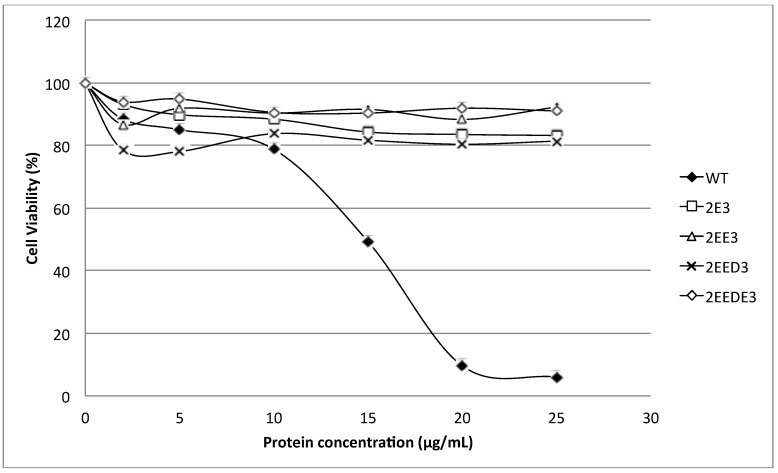
Cytolytic activity of the wild type (WT) and mutants (2E3, 2EE3, 2EED3 and 2EEDE3). A total of 4 amino acids (Glutamic acid-E, Glutamic acid-E, Aspartic acid-D and Glutamic acid-E) were inserted in succession at the *N*-terminus. Mutants generated displayed significantly lower cytotoxic activity compared to the wild type.

The MTT assay was used to assess the cytolytic activity of recombinant HALT-1 and its mutants. HeLa cells were incubated with recombinant HALT-1 at various concentrations (2, 5, 10, 15, 20, 25 μg/mL) for 24 h and the concentration of viable cells was measured by recording the changes in absorbance. As shown in Figure 5, recombinant HALT-1 reduced the viability of HeLa cells in a dose-dependent manner and the cells were completely killed by HALT-1 at a concentration of 20 μg/mL after 24 h of incubation. The IC_50_ value, which is defined as the concentration of recombinant HALT-1 at which 50% of cells die, was found to be approximately 15 μg/mL. By comparison, the IC_50_ of EqtII on lung fibroblasts was reported to be 17 ng/mL after one hour of incubation [13]. The IC_50_ value for EqtII tested on two tumor cell lines, Ehrlich ascites carcinoma and leukemia cells was also very low [14]. Thus, while HALT-1 is clearly cytotoxic, our results showed that it is less potent than equinatoxin EqtII.

**Figure 6:**
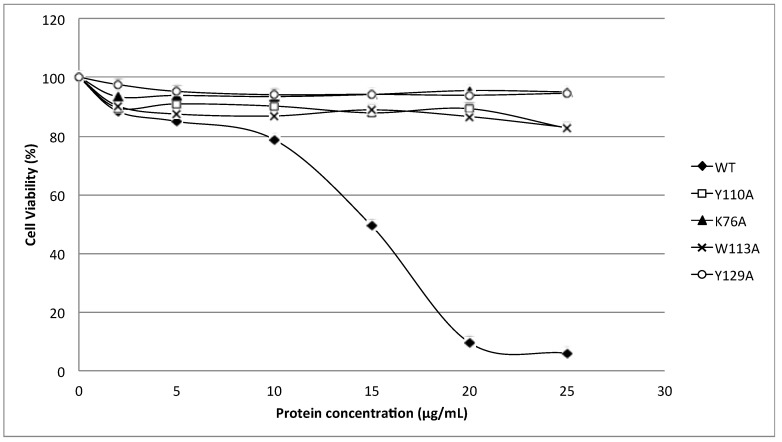
Cytotoxic activity of the wild type (WT) and mutants (Y110A, K76A, W113A and Y129A). Three aromatic amino acids and one lysine were substituted into alanine (A) and all the mutants generated exhibited a significantly lower cytotoxic activity as compared to the wild type.

Figure 5 shows the cytolytic activity of HALT-1 mutants with a lengthened *N*-terminal α-helix (2E3, 2EE3, 2EED3 and 2EEDE3). The mutant proteins showed essentially no cytolytic activity (Figure 5). The viability of HeLa cells was maintained at more than 80% when treated with mutant HALT-1 proteins. The IC_50_ of the wild type HALT-1 was 15 μg/mL under the same conditions. The mutants K76A, Y110A, W113A and Y129A, which disrupt the conserved aromatic cluster involved in membrane binding, also showed nearly no cytolytic activity at all concentrations tested (Figure 6).

## 3. Discussion

The first 20 residues of HALT-1 protein constitute a signal peptide. Unlike other actinoporins such as equinatoxins, the HALT-1 signal peptide is not followed by a propeptide and thus its active form is likely not regulated by the endoproteolytic cleavage. Directly following the HALT-1 signal peptide is the *N*-terminal region, which comprises about 30 amino acids. In this region, amino acids 14 to 25 are predicted to form an α-helix (Figure 1). As all actinoporins are structurally and functionally conserved, we assume that the *N*-terminal α-helix of HALT-1 exhibits an amphipathic nature and can be translocated into the plasma membrane pore [12]. In EqtII, the corresponding α-helix encompasses residues 10 to 28 (Figure 1) and contains both negatively charged aspartic acid (residues 10 and 17) and glutamic acid (residue 24) that greatly facilitates the transfer of its flexible *N*-terminus into the cation selective pores [7]. Evidence has shown that an additional negative charge at the *N*-terminus of EqtII by sulfhydryl modification improved the cationic selectivity and thus increased the conductance of the pore [15]. Nevertheless, our results show that extending the *N*-terminus of HALT-1 with 1–4 negatively charged residues reduced haemolytic and cytolytic activity compared to the wild type (Figure 3 and Figure 5). This could be due to the increased length of the *N*-terminus or the increased negative charge.

Supporting the first idea are the fact that all actinoporins have approximately 30 amino acids forming an amphipathic helix at the *N*-terminus and the fact that removal of five or ten residues from the *N*-terminus of EqtII reduced haemolytic activity and presumably pore formation [16]. Similarly, the addition of 1–4 amino acid(s) at the *N*-terminus of HALT-1 alters the length of the *N*-terminal α-helix and might inhibit pore formation and diminish haemolytic and cytolytic activity. Alternatively, changes in length of the *N*-terminus may disrupt the ability of the *N*-terminal α-helix to dissociate from the β-sheet core of the actinoporin structure during pore formation. The requirement for structural flexibility of the *N*-terminal α-helix was clearly demonstrated in a double cysteine mutant which introduced a disulfide bond and locked the α-helix to the core β-sheet and prevented pore formation [6,9].

Apart from the size constraint, the amphipathic nature in the *N*-terminal region is another important determinant for successful integration of this region into the plasma membrane. An electrophysiological study on the planar lipid bilayer has suggested that when the *N*-terminal region of actinoporins is translocated into the interfacial surface of plasma membrane, it is essential to have the negatively charged amino acids facing the pore lumen, while the polar amino acids are positioned toward the hydrophobic edge [7]. The *N*-terminal region of HALT-1 also contains negatively charged aspartic acids at position 15, 22 and 23, although they are not located at the positions where the negatively charged amino acids are found in other actinoporins. Based on our findings, the addition of one negatively charged residue (E) reduced 80% of haemolytic activity while two or more negatively charged residues (ED or EDE or EEDE) led to further reduction in activity. This indicates that excess negative charges at the end of the *N*-terminus of HALT-1 were unfavorable for haemolytic and cytolytic activity. Interestingly, although both sticholysins StnI and StnII have 93% sequence identity [17], the *N*-terminal region of sticholysin StnI has two additional D and E residues compared to sticholysin StnII and exhibits lower haemolytic activity [18,19,20]. Thus, similar to other actinoporins, HALT-1 requires the *N*-terminus, including the amphipathic α-helix, to efficiently make pores in lipid membranes. However, we do not rule out the possibility that extending the *N*-terminus of HALT-1 resulted in a change of the native structure and thus the loss of protein function.

**Figure 7:**
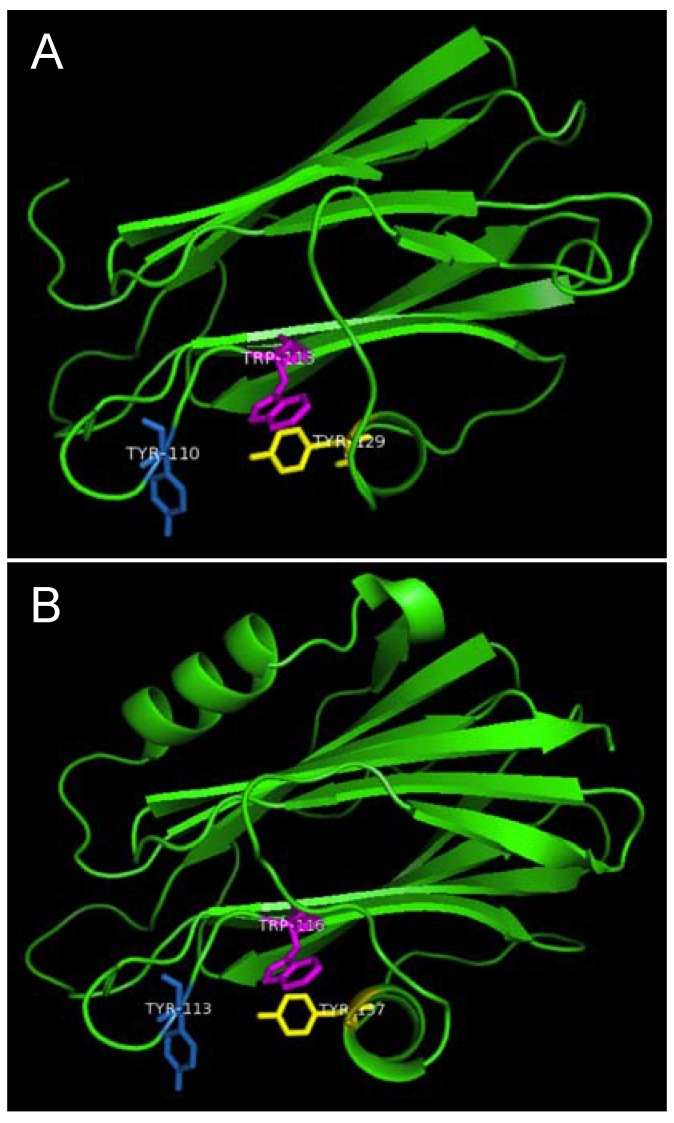

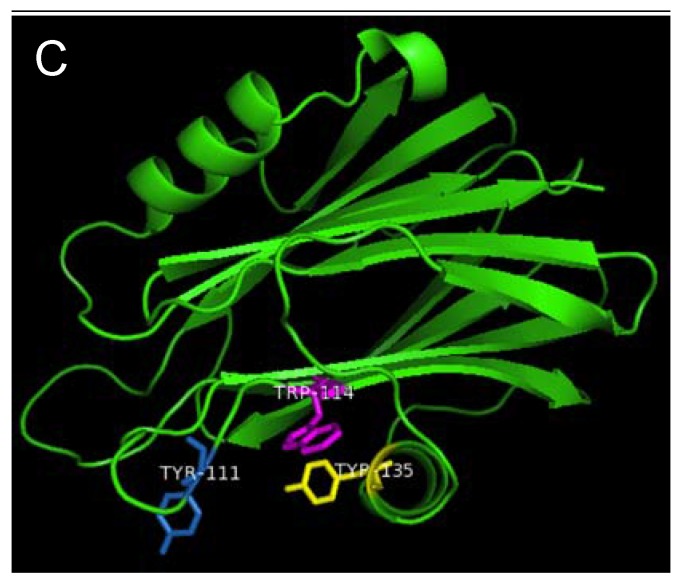
Aromatic amino acid residues of HALT-1, EqtII and StnII. All residues are shown as sticks in different colours. (**A**) Tyr110 (skyblue), Trp113 (magenta) and Tyr129 (yellow) from HALT-1; (**B**) Tyr113, (skyblue), Trp116 (magenta) and Tyr137 (yellow) from EqtII; (**C**) Tyr111 (skyblue), Trp114 (magenta) and Trp135 (yellow) from StnII. All these tyrosine (Tyr) and tryptophan (Trp) are well conserved between HALT-1, EqtII and StnII and they are predicted to form an aromatic patch on surface of the molecule which could be essential for the insertion into water-lipid interface.

Strands β6, β7 and helix α2 of actinoporins contain a conserved cluster of aromatic amino acids and a POC (phosphocholine) binding site which are required for specific recognition of the phosphocholine head group of spingomyelins in the cell membrane [21,22]. The aromatic amino acids, Tyr113, Trp116 and Tyr137 in EqtII and Tyr111, Trp114 and Tyr135 in StnII (Figure 1) have been identified as critical for this binding [22,23]. In HALT-1, the corresponding amino acids are Tyr110 (in strand β8), Trp113 (in strand β8) and Tyr129 (in helix α2) (Figure 1) and they are expected to form a similar aromatic patch on the surface of the molecule as shown in the model in Figure 7. In fact, these three aromatic amino acids are conserved in all actinoporin family members [24]. Substitution of these aromatic amino acids by alanine in HALT-1 mutants Y110A, W113A and Y129A led to an almost complete loss of haemolytic and cytolytic activity (Figure 4 and Figure 6). The loss of activity observed in these three mutants may not be due to structural instability, since mutations at the surface of a protein are usually not deleterious as compared to mutations in the core, and both protein stability prediction tools indicated that the mutations are either neutral or slightly destabilizing. Similar results have been obtained with EqtII. Replacement of Trp116 with phenylalanine reduced the mutant membrane-binding significantly, leading to the loss of haemolytic activity [22]. Replacement of Trp116 with alanine also reduced membrane binding and haemolytic activity [6]. The importance of these aromatic amino acids became more evident when the cocrystal structure of StnII and POC at 2.4 Å resolution revealed that POC bound to a cavity in which its choline moiety interacts with the electron-rich aromatic ring of Tyr111 and Tyr135 [5]. A similar POC-binding cavity is also predicted in HALT-1 whereby the aromatic rings from Tyr110, Trp113 and Tyr129 project into the cavity (Figure 7).

Finally, the K76A mutant also resulted in a decrease of haemolytic and cytolytic activity (Figure 4 and Figure 6). K76 is positioned at the end of strand β5 (Figure 1) and is a conserved residue also found in EqtII (Lys77) and StnII (Lys75). Our result is in good agreement with the results of Anderluh* et al.* [25] showing that lysine 77 of EqtII (Figure 1), when substituted with cysteine, led to the decrease of haemolytic activity although the structure remained intact. Furthermore, the authors claimed that the decrease of haemolytic activity was due to the loss of positive charge and thus the inability of the K77C mutant to oligomerize on lipid membranes. Consistent with this interpretation, the haemolytic activity was almost completely restored when a positive charge was reintroduced at position 77. Further supporting data were derived from the 2D crystallization of StnII with lipid monolayer [5]. In this high resolution model, it clearly showed that K75 (which lies in the loop between β5 and β6) of StnII was in close proximity to lipidic interface, stabilizing the conformation of the toroidal pore. Hence, we can hypothesize that lysine 76 from HALT-1 is also involved in interaction with membrane lipids and in oligomerization and that mutation to an uncharged alanine impairs this interaction.

## 4. Experimental Section

### 4.1. Isolation of HALT-1 cDNA

Total RNA was extracted from approximately 100 mg tissue of *Hydra magnipapillata* strain 105 using TRIzol^®^ Reagent (Invitrogen-Life Technologies, Carlsbad, CA, USA). First strand cDNA was then synthesized according to the manufacturer’s instructions using the SuperScript™ III First-Strand Synthesis System (Invitrogen-Life Technologies, Carlsbad, CA, USA). Subsequently, double-stranded cDNA was generated by the initial PCR with a set of forward and reverse primers (5'TTCACTCACGTTGATTTATACCTT3' and 5'TTGCTCCACTCTTCTATTAGCTC3', respectively) and followed by the nested PCR using another set of forward and reverse primers (5'GGGTAGGACAACTGGAATAGT3' and 5'GCTGACTGCTTGGTGAATAC3', respectively). An amplified cDNA fragment from *HALT-1* was cloned into pCR4-TOPO vector and then transformed into One Shot^®^ TOP10 *E. coli* (Invitrogen-Life Technologies, USA). Verification of the *HALT-1* insert was done through sequencing.

### 4.2. Construction of Expression Vector of HALT-1

To heterologously express HALT-1, its coding region was directionally sub-cloned into an expression vector pET28a (Novagen, EMD biosciences, San Diego, CA, USA) and transformed into BL21(DE3) (New England BioLabs Inc., Ipswich, MA, USA). Briefly, *HALT-1* cDNA with *Sal*I and *Not*I flanking at both ends was constructed with *HALT-1*-pCR4-TOPO and two primers (5'GCAGTCGACGAGCAGCTTTAGGAGTTATA3' and 5'CTTGCGGCCGCCTTTCGAATATTCTTATCC3') containing *Sal*I and *Not*I sites (underlined nucleotides) at the 3' and 5' ends, respectively. This was followed by insertion into pET28a using the LigaFast™ Rapid DNA Ligation System (Promega, Madison, WI, USA). The correct reading frame of the inserted *HALT-1* was confirmed by sequencing before transforming it into BL21(DE3).

### 4.3. Synthesis of HALT-1 Mutants

Site-directed mutagenesis was performed on pET28a and mutations were introduced according to the manufacturer’s instructions (Agilent, Santa Clara, CA, USA). In brief, PCR was carried out with the mutagenic forward and reverse primers using *Pfu* DNA polymerase. PCR was started at 95 °C for 30 s and repeated for 16 cycles at 95 °C for 30 s, 60 °C for 1 min and 68 °C for 5 min. Newly synthesized mutant strands were cooled on ice for 2 min and then subjected to *Dpn*1 digestion to remove the parental strand. The new strand containing the mutation was then transformed into XL1-Blue competent cells. Colonies appeared on the agar plate should contain the mutant clone. The DNA plasmid of the mutant clone was purified using the Wizard Plus SV Minipreps DNA purification system (Promega, Madison, WI, USA) and sent for sequencing before the transformation into BL21(DE3).

### 4.4. Protein Expression and Affinity Purification

His-tag fusion HALT-1 and its mutants were expressed in BL21(DE3) in the presence of IPTG (1 mM) for 3 h at 37 °C with agitation. Each induced culture was then resuspended in the lysis buffer (50 mM NaH_2_PO_4_; 0.5 M NaCl; 10 mM Imidazole; pH 8.0) with 1× Halt Protease Inhibitor Cocktail prior to sonication at 130 watt and 20 kHz for a total of 120 s per sample on ice. The soluble fraction of the crude cell lysate was subjected to the affinity chromatography of nickel-chelating resin, Ni-NTA superflow (Qiagen, Hilden, Germany). The purified His-tag fusion protein was concentrated and exchanged with a 1× phosphate buffered saline (PBS) buffer using an Amicon Ultracel Column (Millipore, Billerica, MA, USA).

### 4.5. Haemolysis Assay

Human erythrocytes were used to assess the haemolytic activity of recombinant HALT-1. Firstly, 2 mL of blood collected from humans were washed three times with sterile 0.85% NaCl saline solution. Erythrocytes were pelleted at 1000 *g* for 5 min at room temperature and supernatant was then carefully discarded upon each wash. The erythrocyte pellet was resuspended with 0.85% NaCl saline solution to obtain 1% erythrocytes. Serial concentrations of either HALT-1 or its mutant proteins (5, 10, 15, 20, 25 and 30 µg/mL) were prepared in a final volume of 200 µL and then added to 1 mL of human erythrocytes. PBS buffer instead of protein sample was used as a negative control. The protein-erythrocytes mixtures were incubated at 37 °C for 2 h with gentle rocking at 50 rpm and then centrifuged at 1000 *g* for 5 min at room temperature. The supernatant was measured at OD 540 nm to determine the release of haemoglobin due to the lysis of erythrocytes.

### 4.6. Cytolysis Assay

Cytolytic activity of recombinant HALT-1 and its mutant was evaluated using the HeLa cancer cell line (ATCC CCL2) from RIKEN, Japan. Cells were seeded at 1 × 10^4^ cell/well in 96-well microtiter plate and incubated at 37 °C, 5% CO_2_ for 24 h. Subsequently, serial concentrations (2, 5, 10, 15, 20, 25 μg/mL) of the recombinant HALT1 protein were added into respective wells. Cells were treated with the recombinant protein at 37 °C for 16 h. Cells incubated with the PBS buffer served as a negative control. MTT (3-(4,5-dimethylthiazol-2-yl)-2,5-diphenyltetrazolium bromide) solution (Sigma-Aldrich, St. Louis, MO, USA) of 50 μL (5 mg/mL) was then added into the wells and the cells were incubated for another 4 h. The purple formazan formed was dissolved with dimethyl sulfoxide (DMSO) and the absorbance readings were measured at 570 nm with a reference wavelength at 630 nm. All assays were done in triplicate.

### 4.7. Protein Sequence Alignment

The amino acid sequences of Equinatoxin II (*Actinia equina*), Sticholysin II (*Stichodactyla* *helianthus*) and Bandaporin (*Anthopleura asiatica*) were retrieved from UniProt (http://www.uniprot.org/) with the accession numbers of P61914, P07845 and C5NSL2, respectively. Multiple sequence alignment of mature proteins including HALT-1, Equinatoxin II, Sticholysin II and Bandoporin were conducted using Clustal Omega software, which is available from the European Bioinformatics Institute (http://www.ebi.ac.uk/Tools/msa/clustalo/). The secondary structure prediction of HALT-1 is performed by the submission of amino acid sequence to the web server of PSIPRED (http://bioinf.cs.ucl.ac.uk/psipred/).

### 4.8. Homology Medeling of HALT-1

The three-dimensional structure of HALT-1 was built through homology-modeling using the SWISS-MODEL workspace in the automated mode [26,27]. The 3D structures of Equinatoxin II (PDB ID: 1iaz) and Sticholysin II (PDB ID: 1gwy) were also obtained from Protein Data Bank in Europe (http://www.ebi.ac.uk/pdbe/). The image of the protein models was viewed using PyMOL v1.6 (Version 1.6, Schrödinger, LLC, New York, NY, USA, 2013) (http://pymol.org/).

### 4.9. Prediction of Mutant Stability

Two prediction methods, SDM (Site-Directed Mutator) and I-Mutant 3.0, were used to measure the protein stability change upon point mutation in K76A, Y110A, W113A, A114W and Y129A [28,29]. Based on a set of a set of conformationally constrained environment-specific substitution tables (ESSTs), SDM utilized the PDB structure of HALT-1 (generated by SWISS model) to calculate the difference in the stability scores for the folded and unfolded state for the wild-type and mutant protein structures, *i.e.*, ∆∆G. The mutant can be grouped into seven classes: highly destabilizing (<−2.0), destabilizing (−2.0≤ & <−1.0), slightly destabilizing (−1.0≤ & <−0.5), neutral (−0.5≤ & ≤0.5), slightly stabilizing (0.5< & ≤1.0), stabilizing (1.0< & ≤2.0) and highly stabilizing (>2.0). We used the sequence-based version of I-Mutant 3.0 to calculate ∆∆G and classified the mutant into neutral (−0.5≤ & ≤0.5), large decrease (<−0.5) and large increase (>0.5).

## 5. Conclusions

The molecular characterization of HALT-1 is currently in its infancy, and to date, only one report on HALT-1 haemolytic properties and sphigomyelin specificity has been published [12]. Of the actinoporin family members that have been identified so far, all are isolated from sea anemones (class Anthozoa). Having discovered homologous actinoporins in *Hydra*, which belong to another class, Hydrozoa, it is of interest to determine the evolutionary constraint of HALT-1. In Glasser *et al.* [12], HALT-1 has been proven to be the member of actinoporin family producing the most important findings, such as exerting haemolytic activity, targeting human cell membranes, and forming a larger pore size than EqtII on the cell membrane. In the present study of HALT-1, we identified those amino acid residues that are likely to play a critical role in pore formation and found that they are, in fact, the conserved residues found in almost all actinoporins. Moreover, our study proposes that, similar to other actinoporins, the length of the *N*-terminus of HALT-1 is constrained to approximately 30 residues. Addition of a single residue in this region destroyed the function of the protein. In addition, HALT-1 likely uses the same aromatic amino acids as other actinoporins for binding to the plasma membrane during pore formation.

While there is a growing list of pore-forming toxins, our findings contribute new information about human cell recognition by actinoporins and the formation of ring-shaped tetramer via the insertion of the *N*-terminal α-helix. Such knowledge is crucial for the potential application of actinoporins in biotherapeutics [30,31]. One excellent example is immunotoxin therapy that serves as a potential anticancer agent [32]. The immunotoxin is produced by fusing a target cell-directed antibody and a membrane-acting toxin. In order to minimize nonspecific binding of immunotoxin to normal cells, amino acids in the binding domain of the toxin need to be either modified or eliminated. Thus, by collecting knowledge about functional roles of amino acids in HALT-1 and their structural properties via the mutational studies, one can delete the sphingomyelin binding site from HALT-1 and enable the toxin, as a part of immunotoxin, to eventually recognize the specific target cells through the antibody moiety rather than normal cells.

## Supplementary Materials

Supplementary materials can be accessed at: http://www.mdpi.com/2072-6651/7/2/0407/s1.
